# Supplementary material for: Evaluation of Anti-S1 IgA Response to Different COVID-19 Vaccination Regimens
Source: Vaccines (Basel). 2023 Jun 19;11(6):1117. doi: 10.3390/vaccines11061117 (PMC10301034; doi:10.3390/vaccines11061117)
Supplement: Supplementary file 1 [file vaccines-11-01117-s001.zip › vaccines-2442832-supplementary.pdf]

Supporting information

Table S1. Primary two-dose homologous and heterologous vaccination guidelines.

Vaccine	Dose 1	Dose 2
CoronaVac/ CoronaVac	0.5 mL (3 μg) IM	0.5 mL (3 μg) IM,<br>3–4 weeks
AZD1222/ AZD1222	0.5 mL (5 × 10^{10} IU) IM	0.5 mL (5 × 10^{10} IU) IM,<br>8–12 weeks
CoronaVac/ AZD1222	0.5 mL (3 μg) IM	0.5 mL (5 × 10^{10} IU) IM,<br>3–4 weeks
AZD1222/ CoronaVac	0.5 mL (5 × 10^{10} IU) IM	0.5 mL (3 μg) IM,<br>8–12 weeks
CoronaVac/ BNT162b2	0.5 mL (3 μg) IM	0.3 mL (30 μg) IM,<br>3–4 weeks
BNT162b2/ BNT162b2	0.3 mL (30 μg) IM	0.3 mL (30 μg) IM,<br>3–4 weeks

Abbreviations: IM–intramuscular injection, ID–intradermal injection, IU–infectious unit.

Table S2. The booster dose vaccination plan used in this study.

Vaccine	Dose 3	Dose 4
CoronaVac/ CoronaVac/ BBIBP-CorV	0.5 mL (4 μg) IM,<br>> 3 months	none
CoronaVac/ CoronaVac/ AZD1222	0.3 mL (30 μg) IM<br>≥ 3 months	none
CoronaVac/CoronaVac/ AZD1222(ID)	0.1 mL (10^{10} IU) ID<br>≥ 3 months	none
CoronaVac/ CoronaVac/ BNT162b2	0.3 mL (30 μg) IM<br>≥ 3 months	none
CoronaVac/ CoronaVac/ BNT162b2(ID)	0.1 mL (10 μg) ID<br>≥ 3 months	none
AZD1222/ AZD1222/ BNT162b2/ BNT162b2	0.3 mL (30 μg) IM<br>≥ 3 months	0.3 mL (30 μg) IM<br>≥ 4 months
AZD1222/ AZD1222/ mRNA-1273/ 50 μg mRNA-1273	0.5 mL (100 μg) IM<br>≥ 3 months	0.25 mL (50 μg) IM<br>≥ 4 months

Abbreviations: IM–intramuscular injection, ID–intradermal injection, IU–infectious unit.

Table S3. Characteristics of a subgroup of participants who received three doses of COVID-19 vaccine and were followed up at days 90 to 120.

Characteristics	SV/SV/SP	SV/SV/AZ	SV/SV/PF
Number	23	23	23
Sex, Female no. (%)	11 (47.8)	15 (65.2)	9 (23.1)
Age years, mean [SD]<br>(min–max)	43.8 [7.7]<br>(31.0–64.0)	44.4 [9.9]<br>(20.0–62.0)	41.6 [8.3]<br>(25.0–55.0)
Interval between 1st and 2nd dose (days)<br>Median<br>[IQR]<br>(min–max)	21.0<br>[21.0–24.0]<br>(18.0–30.0)	21.0<br>[21.0–22.0] (17.0–30.0)	27.0<br>[21.0–27.0]<br>(18.0–30.0)
Interval between 2nd and 3rd dose (days)<br>Median<br>[IQR]<br>(min–max)	168.0<br>[164.0–174.0]<br>(115.0–188.0)	148.0<br>[141.0–156.0]<br>(130.0–191.0)	143.0<br>[139.5–147.5]<br>(120.0–160.0)
Interval between the last dose and blood collection at 28 days (days)<br>Median	28.0	28.0	28.0

[IQR]<br>(min–max)	[28.0–30.0]<br>(25.0–35.0)	[28.0–28.0]<br>(22.0–29.0)	[28.0–28.0]<br>(28.0–30.0)
Interval between the last dose and blood collection at 90-120 days (days)<br>Median<br>[IQR]<br>(min–max)	92.0<br>[91.3–92.0]<br>(89.0–113.0)	120.0<br>[120.0–120.0]<br>(119.0–121.0)	120.0<br>[120.0–121.0]<br>(119.0–127.0)

Abbreviations: AZ–AZD1222; ID–intradermal injection; IQR–Interquartile range; PF–BNT162b2; SD–Standard deviation; SP–BBIBP-CorV; SV–CoronaVac.
